# The Rat Pest

**Published:** 1921-04-16

**Authors:** 


### The Rat Pest.

The public has so long regarded the rat with nothing but contempt that it is difficult now to treat with the necessary seriousness one of the really grave dangers to health. An Act of Parliament has been passed, making the occupier of infested premises responsible for a fine up to £5 if he does not take steps to do away with the nuisance. It was preceded by an Order of 1918, which made the owner of the premises liable, but the fact that neither Order nor Act has made any real impression on the public upholds the contention that Acts of Parliament in themselves are useless without some driving force behind them.

Elaborate methods have been discussed for exter-

**THE PUBLIC WELL-BEING**—(*continued*).

minating rats. Probably the most important at this stage is that those with a concern for public economy and the public health should be awakened and should awaken others to the nature of the danger and to the fact that it can be adequately dealt with. The damage done almost passes the belief of the ordinary person who has never or rarely seen a rat. It is not merely the food they eat which is destroyed, but the food of the same kind which is ruined by their contamination, and, in addition to that, food which rats do not normally eat is ruined by their passage through it. Recently in one large warehouse a large consignment of tomatoes had to be destroyed because the rats had been at it.

The normal shrinking from the rat comes largely from the fact that he has always been associated with uncleanness. His own immunity from the diseases he carries has added enormously to his capacity for damage. The discovery of the part played by the rat-flea in carrying the germ of plague from rat to man has explained his immunity and displayed at the same time the tremendous multiplication of the risk which this fact entails. Other diseases pass from the rat to human beings by way of the pig, and Japanese observers have recently shown that the parasites of protozoal diseases are found in rats and probably conveyed to man by means of fleas and lice, and, incidentally, they have thus reminded us that the rat is a world pest and is not likely to cease to cost us both in money and in life until he becomes as extinct as the Dodo. To regard such a task as impossible is as unscientific as to regard it as easy. The disappearance of animals is largely a question of environment, and it is to the interest of humanity in more ways than one that the environment in which rats thrive should be altered, and in this view the rat is not merely a danger in himself, but a pointer to other dangers, perhaps in their way even more serious. This, as shown by an exhaustive report from Manchester, is the basis of the new methods of dealing with the rat plague which are being worked out by many local authorities, with the help of official rat officers. They find th[illegible] where the rats are driven out of premises which are at the same time made ratproof the evil is practically at an end, whereas in premises where the old-fashioned methods of chasing the rats from one building to another are relied on, an almost definite date for their reappearance in numbers can be fixed.

Many business premises are found to have old, forgotten drains running right into them. In other buildings the rat's gateways are found to be holes in the walls or cavities open for years for the deposit of dust. Such conditions are in themselves unhygienic, whether in buildings used for food storage or as workrooms. In remedying them is found the surest method of carrying out a successful war on the rat. When they disappear the rat goes too, and with him there passes a host of possibilities of insanitariness and disease.

